# EphA4 receptor regulates outwardly rectifying chloride channel in CA1 hippocampal neurons after ischemia-reperfusion

**DOI:** 10.1097/WNR.0000000000001311

**Published:** 2019-08-21

**Authors:** Jianguo Li, Na Ma, Jing Chen, Deping Yan, Qian Zhang, Jinchao Shi

**Affiliations:** Key Laboratory of Cellular Physiology, Ministry of Education, Department of Physiology, Shanxi Medical University, Taiyuan, China

**Keywords:** chloride channel, EphA4, ephrinA3, hippocampal neurons, ischemia-reperfusion

## Abstract

CA1 hippocampal neurons are sensitive to ischemia. The erythropoietin-producing hepatocellular carcinoma (Eph) receptors are a cell-cell contact signaling pathway for regulating neuron function and death. However, the mechanisms of EphA receptor in neuron death after ischemia remain unclear. In this study, we present evidence that outwardly rectifying chloride channels reside in CA1 hippocampal neurons. EphA4 receptor increased chloride channel currents. Moreover, the EphA4 receptor no longer had significant effects on enhanced channel currents following ischemia-reperfusion. Inhibition of EphA4 receptor with EphA4-Fc significantly decreased the channel currents after ischemia-reperfusion. These results suggest that the increased effect of the EphA4 receptor on the outwardly rectifying chloride channel activity in CA1 hippocampal neurons may provide better treatment for ischemic brain injury.

## Introduction

Interruptions in the blood supply to the brain lead to ischemic stroke, which is the leading cause of chronic disability and the third leading cause of death in humans [1]. Despite many research on the ischemic neuronal death, there is limited information about the exact cellular mechanisms. The only effective treatment for ischemic strokes at present is reinstating a blood supply [2].

Pyramidal neurons in the CA1 region of rat hippocampus are particularly vulnerable to ischemic insult and die about 3 days after transient forebrain ischemia, a process named delayed neuronal cell death [3]. A number of studies suggest that this ischemic delayed neuronal death involves apoptosis [4]. There are multiple mechanisms, such as death receptor and receptor tyrosine kinases (RKTs) activation, for the induction of apoptosis [5].

Signals following cell-cell interaction provide important means for regulating normal neural function and survival [6]. One group of the contact-dependent pathways includes the erythropoietin-producing hepatocellular carcinoma (Eph) receptors and ephrin ligands signaling pathway. Eph receptors constitute the largest family of RKTs in mammalians and are subdivided into A and B classes. EphA and EphB family receptors are activated generally by ephrinA or ephrinB ligands, respectively [7]. EphA4 and ephrinA3 are expressed at high levels in the hippocampus, particularly in the CA1 region [8]. EphA receptors regulate function and plasticity in the hippocampus [9]. Transient forebrain ischemia increased the expressions of ephrinA3 and EphA4 in the CA1 region. Blocking the EphA4 receptor with EphA4-Fc attenuated apoptotic neuronal cell death [10]. Although an increasing amount of evidence supports a role for these molecules in apoptosis, the mechanisms of EphA receptor signaling in neuronal cell death have been not studied in detail [11].

Over-activation of Cl^−^ efflux pathways that mediate apoptosis volume decrease has also been involved in the induction of apoptosis [12,13]. 4,4′-diisothiocyanatostilbene-2,20-disulfonic acid (DIDS), a chloride channel blocker, attenuated the ischemic neuronal death in rat CA1 hippocampal neurons [14]. However, modulations of the outwardly rectifying chloride channel in adult rat hippocampal neurons are still unclear. Some evidence revealed that RKTs may modulate chloride channels [15].

Therefore, in the present study, we investigated the effects of EphA4 receptor on the outwardly rectifying chloride channel in the hippocampal CA1 region of adult rats in a transient forebrain ischemia model. These results suggest a novel mechanism for modulating the outwardly rectifying chloride channel in CA1 hippocampal neurons after transient ischemia, and that may provide a therapeutic target for the treatment of stroke.

## Material and methods

### Animals and hippocampal slices preparation

All of the experiments were performed on male adult Wistar rats (8–9 weeks) weighing 180–220 g. Food and water were ad libitum available. All experimental procedures were carried out in accordance with the Guide for the Care and Use of Laboratory Animals in China. Rats were anesthetized with chloral hydrate (i.p., 35 mg/100 g weight) and decapitated. Brains were quickly removed, iced, and blocked for slicing. The blocked tissue was cut into 400 μm thick slices with a Vibratome3000 (Vibratome, St Louis, MO, USA) whereas bathed in a low Ca^2+^, HEPES-buffered solution containing (in mmol/L) sodium isethionate 140, KCl 2, MgCl_2_ 4, CaCl_2_ 0.1, glucose 23, and HEPES 15. Slices were then incubated in NaHCO_3_-buffered saline bubbled with 95% O_2_/5% CO_2_ containing (in mmol/L) NaCl 125, KCl 2.5, CaCl_2_ 2, MgCl_2_ 2, NaHCO_3_ 26, NaH_2_PO_4_ 1.25, and glucose 10. pH was adjusted to 7.4 with NaOH.

### Whole-cell patch-clamp experiments

All experiments in hippocampal slices were conducted at 37°C. Whole-cell voltage-clamp recordings were made from the pyramidal neurons in the CA1 region that were visually identified by their location using an upright microscope. Membrane currents were recorded using an Axopatch 200B amplifier (Molecular Devices, Sunnyvale, California, USA). The neurons were voltage-clamped at −40 mV and the currents were evoked by depolarizing voltage steps from −100 to +40 mV with an increment of 20 mV. Currents were filtered at 1 kHz and digitized at 10 kHz. Data acquisition and analysis were carried out using pCLAMP (Molecular Devices). The patch electrode had a resistance of 3.5–5 MΩ when filled with pipette solution. The isotonic bath solution contained (in mmol/L) N-Methyl-D-glucamine Chloride (NMDG-Cl) 140, MgCl_2_ 2, HEPES 10, glucose 10, 4-AP 2, EGTA 1. DIDS was added to block the outwardly rectifying chloride channel. The pipette solution contained (in mmol/L) NMDG-Cl 25, NMDG 115, Aspartic acid 100, MgCl_2_ 2, EGTA 1, HEPES 10, Na_2_ATP 5. pH was adjusted to 7.4 with NMDG-OH.

### Transient forebrain ischemia

Transient forebrain ischemia was induced by the use of the four-vessel occlusion method with some modification [16]. Briefly, rats were anesthetized with chloral hydrate. Both vertebral arteries were electrocauterized permanently, and both common carotid arteries were exposed for subsequent occlusion. On the following day, rats were restrained, and the carotid clasps were tightened to produce four-vessel occlusion. Severe transient forebrain ischemia was induced by occluding both common carotid arteries for 15 minutes. Cerebral blood flow was resumed immediately after the release of carotid artery clasps. The following indicators of forebrain ischemia were monitored: unresponsiveness, loss of righting reflex and catatonic postures. Rats with post-ischemic convulsions were excluded from the study. Rectal temperature was continuously monitored and kept at 37°C with a heating pad.

### Statistical analyses

All data were expressed as the mean ± SEM. Unpaired Student’s *t*-test and one-way analysis of variance with Student–Newman–Keuls *post hoc* test were used for statistical analysis. Values of *P* < 0.05 were considered statistically significant.

## Results

### Outwardly rectifying chloride channel in rat CA1 hippocampal neurons

**Fig. 1:**
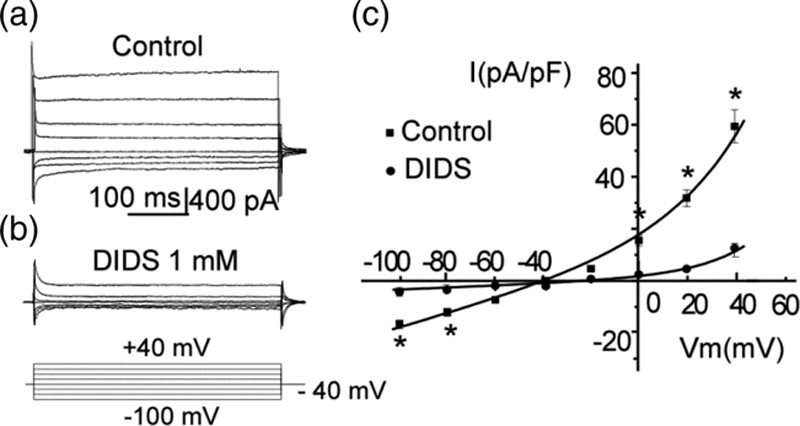
The outwardly rectifying chloride channel in rat CA1 hippocampal neurons. (a) Original current traces from hippocampal slices. Traces were recorded using the whole-cell patch-clamp configuration. The neurons were voltage-clamped at −40 mV and the traces were evoked by depolarizing voltage steps from −100 to +40 mV with an increment of 20 mV (see inset at the bottom of panel b). (b) Original traces show the chloride channel block effects of DIDS. (c) Corresponding current-voltage relationship of the channels treated with (•) or without (▪) 1 mM DIDS in the bath solutions. The shape of the best-fit curve indicates an outward rectification pattern. Data are the mean ± SEM. n = 6; *P < 0.05 versus controls. DIDS, 4,4′-diisothiocyanatostilbene-2,20-disulfonic acid.

Functional expression of outward rectifier chloride channel in CA1 hippocampal neurons was identified with whole-cell recordings configuration from hippocampal slices (Fig. 1a). DIDS (1 mM) significantly blocked the Cl^−^ currents (Fig. 1b and c) (n = 6, *P* < 0.05). Whole-cell currents and the current-voltage relationship exhibits an outward rectification pattern (Fig. 1c). The activated currents were Cl^−^ in nature because the reversal potential was nearly equal to the equilibrium potential of Cl^−^ (−40 mV) but not of any other ions. Major cations in the experimental solutions were replaced by NMDG.

### EphA4 receptor activation increases outwardly rectifying chloride channel currents in CA1 hippocampal neurons

**Fig. 2:**
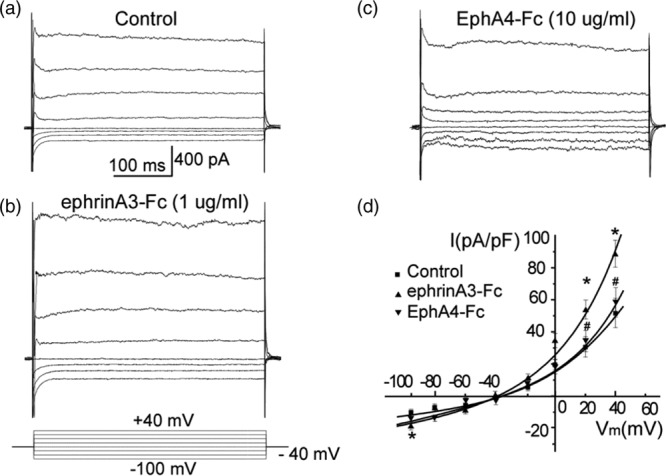
EphrinA3-Fc increases the activity of the outwardly rectifying chloride channel in CA1 hippocampal neurons. (a) Original traces show outward rectifier chloride channel from CA1 hippocampal neurons. Traces were recorded using the whole-cell patch-clamp configuration. The neurons were voltage-clamped at −40 mV and the traces were evoked by depolarizing voltage steps from −100 to +40 mV with an increment of 20 mV (see inset at the bottom of panel b). (b) Original traces show increased Cl^−^ currents after treating the hippocampal slices with ephrinA3-Fc. (c) Original traces show the currents were partly recovered by EphA4-Fc, an EphA4 receptor inhibitor. (d) Corresponding current-voltage relationship of chloride channels in control (▪), treated with ephrinA3-Fc (▴), or EphA4-Fc (▾) in the bath solution. Data are the mean ± SEM. n = 6; *P < 0.05 ephrinA3-Fc versus controls; #*P* < 0.05 EphA4-Fc versus ephrinA3-Fc.

Slices were treated with ephrinA3-Fc to activate the EphA4 receptor. EphrinA3-Fc (1 μg/ml) in the bath solution led to increased Cl^−^ currents (Fig. 2a and b). Subsequent treatment with EphA4-Fc (10 μg/ml), an EphA4 receptor antagonist, in the bath solution led to a significant recovery of the channel activity (Fig. 2c and d) (n = 6, *P* < 0.05).

### Effects of EphA4 receptor activation on the outwardly rectifying Chloride channel in CA1 hippocampal neurons after ischemia-reperfusion

**Fig. 3:**
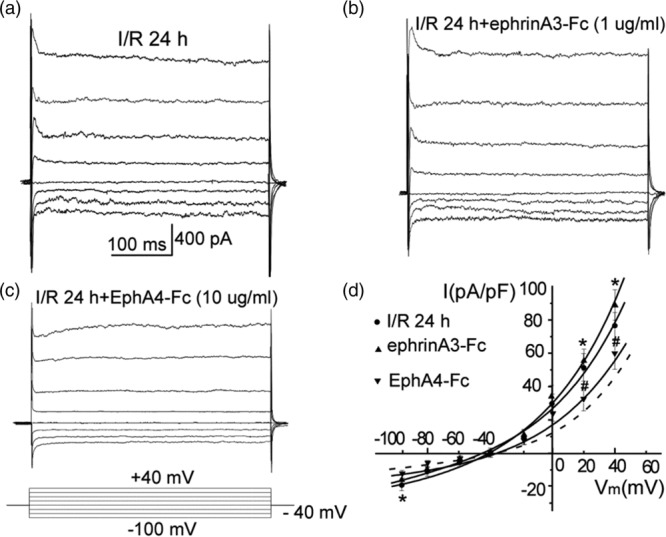
Effects of EphA4 receptor activation on the outwardly rectifying chloride channel in CA1 hippocampal neurons after ischemia-reperfusion (I/R). (a) Original traces show Cl^–^ currents at 24 hours after I/R. Traces were recorded using the whole-cell patch-clamp configuration. The neurons were voltage-clamped at −40 mV and the traces were evoked by depolarizing voltage steps from −100 to +40 mV with an increment of 20 mV (see inset at the bottom of panel c). (b) Original traces show decreased currents in the postischemic neurons after treating with ephrinA3-Fc. (c) Original traces show the currents in the post-ischemic neurons after treating with EphA4-Fc. (d) Corresponding current-voltage relationship of chloride channels at 24 hours after I/R (▪), treated with ephrinA3-Fc (▴), or EphA4-Fc (▾) in the bath solution. The dashed current-voltage curve represents the normal control. Data are the mean ± SEM. n = 6; *P < 0.05 24 hours after I/R versus controls; #*P* < 0.05 EphA4-Fc versus I/R. I/R, ischemia-reperfusion.

Figure 3a shows original traces of chloride channel currents in CA1 hippocampal neurons. This indicated increased chloride channel activity at 24 hours after reperfusion following 15 minutes of ischemia (Fig. 3a and d) (n = 6, *P* < 0.05). To elucidate the role of EphA4 receptor signaling in ischemia-induced over-activity of chloride channel, ephrinA3-Fc was used to activate EphA4 receptor. Treatment with ephrinA3-Fc no longer had a significant effect on the Cl^−^ currents (Fig. 3b and d). These enhanced chloride currents; however, were decreased by inhibiting EphA4 receptor with EphA4-Fc (10 μg/ml) (Fig. 3c and d) (n = 6, *P* < 0.05). These data suggest EphA4 receptor activation increased the Cl^−^ currents after ischemia-reperfusion (I/R).

## Discussion

In the present study, we demonstrated that EphA receptor potentiated the activity of outwardly rectifying chloride channel in rat hippocampal neurons after I/R.

EphrinA/EphA cell-cell contact signaling pathway plays an important role in the apoptosis of CA1 hippocampal neurons induced by ischemia [10]. The expression of ephrinA3 and EphA4 was increased in the CA1 region following I/R. Blocking the EphrinA3/EphA4 interaction attenuated apoptotic neuronal cell death through the inhibition of caspase-3 activation [10].

To study the mechanism that may underlie the EphA receptor involvement in cell death, we recorded an outwardly rectifying chloride channel that induces neuronal cell death [17]. Although treatment with ephrinA3-Fc to activate the EphA4 receptor increased the amplitude of chloride channel currents under control condition, it had no significant effects on the enhanced activity of the chloride channel after I/R. This revealed that the channel activity in post-ischemic neurons has already been modulated by EphA4 receptor. In addition, the evidence that EphA4 inhibitor treatment decreased the chloride currents in control and I/R conditions supported the enhancement effects of the EphA4 receptor. These results indicated that the EphA4 receptor leads to enhanced activity of outwardly rectifying chloride channel in adult rats CA1 hippocampal neurons following ischemic injury.

Cell volume change caused by Cl^-^ efflux via chloride channels is an important mechanism of ischemic neuronal death. As a major hallmark of apoptosis, apoptosis volume decrease is an early prerequisite to apoptotic events and is associated with activation of chloride and potassium channels [18,19]. Chloride channel blocker DIDS or potassium channel blocker tetraethylammonium attenuated the ischemic neuronal death in the CA1 hippocampus of rats [14]. Thus, EphA4-Fc may attenuate neuronal cell death by maintaining the cell volume via inhibiting chloride channel activity.

EphA RKT activation can induce a variety of signaling pathways that may modulate the activity of ion channels, such as Src family kinases signaling, mitogen-activated protein kinase signaling, and Ephexin signaling to the small GTPases RhoA, Rac, or Cdc42 [20]. EphA receptor can activate voltage-sensitive calcium channels via activating Cdc42 [21]. Treatment of cells with ephrinB2 led to N-methyl-D-aspartate (NMDA) channel phosphorylation through the activation of the Src family of tyrosine kinase [22]. The tyrosine phosphorylation of α1 subunits by Src family kinase contributed to the upregulation of L-calcium channel activity in the post-ischemic hippocampus [23]. Activation of MAPK is a critical step in the opening of chloride channels. Given that inhibitors of tyrosine kinase suppress chloride channel activity in a variety of cell types [24], EphA4 receptor may modulate the outwardly rectifying chloride channel via activating its multiple downstream signaling pathways. Since the molecular identity of this chloride channel has not been elucidated, we cannot rule out the direct function of the Eph receptor via colocalization with outwardly rectifying chloride channel, that phenomenon was found between Eph receptors and calcium channels or NMDA channels at the neuronal cell surface [25].

We cannot exclude possibilities for the roles of the EphA4 receptor in the neuronal cell death through other mechanisms. Studies have shown that upregulated expression of ephrinA3 and EphA4 in the hippocampus reduces the glial glutamate transporters that transport the increased glutamate induced by ischemia into the glia [9]. The EphA4 receptor is able to induce apoptosis in adult subventricular zone independent of the phosphorylation function of the receptor [26].

## Conclusion

These findings suggest that EphA4 receptor activation increases the activity of outwardly rectifying chloride channel in the CA1 hippocampal neurons after I/R. These may contribute to the pro-apoptotic effects of EphA4 receptor and provide better treatments of ischemic brain injury.

## Acknowledgements

This work was supported by National Natural Science Foundation of China (No. 81671231), Shanxi Scholarship Council of China (No. 2017–056) and the Fund for Shanxi ‘1331 Project’ Key Subjects Construction (XK201708).

## Conflicts of interest

There are no conflicts of interest.
